# Supplementary material for: Effect of organizational change on employee innovation performance: A dual mediation model
Source: PLoS One. 2025 Feb 10;20(2):e0313056. doi: 10.1371/journal.pone.0313056 (PMC11809867; doi:10.1371/journal.pone.0313056)
Supplement: Attachment — Submitted filename: Response to Reviewers.docx [file pone.0313056.s002.docx]

We sincerely thank you to your valuable feedback that we have used to improve the quality of our manuscript. According to your nice suggestions, we have made changes to our previous draft and the changes/additions to the manuscript are given in the red text.

**Response to Reviewer #1:**

1. **Regarding the revision of the abstract:**

Dear reviewer, thank you for your valuable suggestions. We have reorganized the first sentence of the abstract, breaking it into two sentences to improve clarity and readability.

2. **Description of research subjects and analytical techniques:**

Thank you for your suggestion. We have provided a detailed description of the research subjects in the methods section, including the background, organization, and country of the participants. At the same time, we have clearly explained the analytical techniques and tools used, as well as the contribution of these choices to the significance of the research.

3. **Consistency in variable naming:**

We have standardized the terminology used throughout the text for "employee innovation performance" and "company innovation performance" to ensure consistency and accuracy throughout the study.

4. **Citations in the introduction section:**

We have added more relevant literature citations in the introduction section, especially in the areas of JD-R model, job stress, job engagement, and organizational identification, to strengthen our arguments and the background of the research.

5. **Enrichment of theoretical background and hypotheses:**

We have updated the theoretical background and hypotheses section, including the latest relevant literature, especially in the application and interpretation of the JD-R model, to ensure the modernity and relevance of the theoretical framework.

6. **Consistency of terminology:**

We have checked the entire text to ensure the consistency of terms such as "job demands" and "job resources", avoiding the use of inconsistent terminology to reduce confusion for readers.

7. **Explanation of data analysis procedures:**

In the methods section, we have detailed the steps and tools used for data analysis to enhance the transparency and credibility of the research.

8. **Completeness of the results section:**

We have checked and ensured that all mentioned tables, including Table 5, are included in the results section to ensure the completeness of the research findings.

9. **Citations in the discussion section:**

We have added necessary citations in the discussion section to ensure that all discussions are supported by literature and to avoid any unsubstantiated statements.

10. **Introduction of social identity theory:**

We have introduced social identity theory earlier in the theoretical background and hypotheses section to ensure the completeness and coherence of the theoretical framework.

**Response to Reviewer #2:**

1. **Industry types of the interviewee's companies:**

Thank you for the reviewer's suggestion. We have clearly stated in the methodology section the industry types of the 5 companies where the interviewees are located, and discussed how these industry types are related to organizational change needs and innovation performance, as well as how these factors affect organizational change and innovation performance.

2. **Discussion on model explanatory power:**

We have detailed the implications of an R2 value below 0.5 in the discussion section, as well as its impact on our model results. Although the R2 value is relatively low, such results are acceptable in social sciences and organizational behavior disciplines considering the specific context of the study, sample size, choice of variables, and other factors. Furthermore, we discussed the significance of the model's explanatory power for the research results, as well as other variables or factors that may need to be considered in practical applications.

3. **Number of samples in Bootstrap test:**

In the methodology section, we explained why we chose 5000 as the number of samples for the Bootstrap test. This usually involves statistical considerations, such as ensuring the stability of the results and reducing sampling errors. In statistics, the number of samples for the Bootstrap method is typically chosen between 1000 and 10000, as this range can provide sufficiently accurate estimates while taking into account computational costs and time.

We greatly appreciate the valuable opinions and suggestions from each reviewer, which have greatly helped us improve the quality of our research. We believe that with these revisions, our study is now more rigorous, clear, and its contribution to academia is more evident.
