# Supplementary material for: Effect of organizational change on employee innovation performance: A dual mediation model
Source: PLoS One. 2025 Feb 10;20(2):e0313056. doi: 10.1371/journal.pone.0313056 (PMC11809867; doi:10.1371/journal.pone.0313056)
Supplement: Attachment — Submitted filename: Response to Reviewers.docx [file pone.0313056.s003.docx]

Dear Reviewer #1 and Reviewer #2,

First and foremost, we would like to extend our sincere gratitude for the valuable feedback and suggestions provided. Your insights are instrumental in enhancing the quality of our manuscript. Below are our specific responses to the issues and recommendations you have raised:

**Response to Reviewer #1:**

1. **Regarding the explanation of Model 3:**
   - We apologize for the omission of Model 3. Model 3 is actually an intermediate model designed to introduce control variables and establish a foundation for further analysis. We will provide a detailed explanation of the composition of Model 3 and its role in the overall analysis in the revised manuscript.
2. **Regarding the display of interaction term regression coefficients:**
   - You are correct in pointing out the insufficient display of interaction term regression coefficients. We will add a table in the results section that lists all interaction term regression coefficients to strengthen the persuasiveness of our arguments.
3. **Regarding statistical evidence for moderation effects:**
   - We acknowledge the lack of sufficient statistical data to support the "moderation effect test" and the "overall moderation mediation model". We will supplement the necessary statistical results to ensure that all moderation effects are adequately supported by data.
4. **Regarding the revision of Figure 1:**
   - Thank you for your suggestions regarding Figure 1. We will adjust the position of organizational identity according to your insights and ensure it correctly reflects its position as an independent variable. We will also refer to the research model you have created for corrections.

**Response to Reviewer #2:**

1. **Regarding the abbreviation error of VUCA:**
   - We apologize for the error in the VUCA abbreviation. We will immediately correct it to "Volatility, Uncertainty, Complexity, Ambiguity" and provide a detailed discussion of VUCA in the discussion and implications section.
2. **Regarding an in-depth discussion of VUCA:**
   - We will add an in-depth analysis of VUCA in the discussion section, exploring its specific impact on our research findings and discussing how to formulate effective strategies in a VUCA environment.

We are committed to addressing all the above issues in the revised manuscript and ensuring the quality and integrity of the paper. We appreciate your time and effort and look forward to your further feedback.

Yours sincerely,

The authors.
